# Supplementary material for: High Performance Thin Layer Chromatography (HPTLC) Analysis of Anti-Asthmatic Combination Therapy in Pharmaceutical Formulation: Assessment of the Method’s Greenness and Blueness
Source: Pharmaceuticals (Basel). 2024 Jul 29;17(8):1002. doi: 10.3390/ph17081002 (PMC11357029; doi:10.3390/ph17081002)
Supplement: Supplementary file 1 [file pharmaceuticals-17-01002-s001.zip › pharmaceuticals-2977608-supplementary.pdf]

High Performance Thin Layer Chromatography (HPTLC) Analysis of Anti-Asthmatic Combination Therapy in Pharmaceutical Formulation: Assessment of the Method's Greenness and Blueness

Huda Salem AlSalem [1], Faisal K. Algethami [2], Maimana A. Magdy [3], Nourudin W. Ali [3], Hala E. Zaazaa [4], Mohamed Abdelkawy [4], Maha M. Abdelrahman [3] and Mohammed Gamal [3,*]

[1] Department of Chemistry, College of Science, Princess Nourah Bint Abdulrahman University, P.O. Box 84428, Riyadh 11671, Saudi Arabia; husalsalem@pnu.edu.sa
[2] Department of Chemistry, College of Science, Imam Mohammad Ibn Saud Islamic University (IMSIU), P.O. Box 90950, Riyadh 11623, Saudi Arabia; falgethami@imamu.edu.sa
[3] Pharmaceutical Analytical Chemistry Department, Faculty of Pharmacy, Beni-Suef University, Alshaheed Shehata Ahmad Hegazy St., Beni-Suef 62514, Egypt; maimanamagdy@yahoo.com (M.A.M.); nour747ali@yahoo.com (N.W.A.); maha_m_abdelrahman@yahoo.com (M.M.A.)
[4] Pharmaceutical Analytical Chemistry Department, Faculty of Pharmacy, Cairo University, Kasr-El-Aini, Cairo 11562, Egypt; hala.zaazaa@pharma.cu.edu.eg (H.E.Z.); dr.mohabdelkawy@hotmail.com (M.A.)
* Correspondence: mgamalm3000@yahoo.com

Table S1: Detailed results of accuracy for the assay of pure samples of HYX, EPH, and THP using the novel HPTLC-densitometric approach.

HYX			EPH			THP		
Taken µg/band	Found* µg/band	Recovery %	Taken µg/band	Found* µg/band	Recovery %	Taken µg/band	Found* µg/band	Recovery %
0.30	0.305	101.67	3.00	2.96	98.67	0.30	0.307	102.33
0.50	0.494	98.80	7.00	6.99	99.86	0.50	0.511	102.20
0.90	0.912	101.33	9.00	8.95	99.44	0.90	0.894	99.33
1.30	1.311	100.85	11.00	11.12	101.09	1.30	1.303	100.23
1.50	1.507	100.47	13.00	13.15	101.15	1.50	1.513	100.87
1.70	1.698	99.88	15.00	15.24	101.6	1.70	1.692	99.53
0.30	0.305	101.67	3.00	2.96	98.67	0.30	0.307	102.33
Mean		100.50	Mean		100.30	Mean		100.75

*Average of 3 determinations

Table S2: Determination of HYX, EPH, and THP in Bronchaline® tablets using the novel HPTLC approach and application of standard addition protocol.

**Pharmaceutical formulation**	component	Taken (µg/band)	Found% * ± SD	Standard addition technique		
				Pure added (µg/band)	Pure found** (µg/band)	Recovery %
**Bronchaline® tablets (Batch No. 14311W) labeled to contain 10 mg of HYX, 15 mg of EPH and 120 mg of THP**	HYX	0.60	96.67 ± 1.429	0.40	0.407	101.75
				0.60	0.592	98.67
				0.80	0.804	100.50
				Mean ± SD		100.31±1.549
	EPH	4.00	95.75 ± 1.249	2.00	1.98	99.00
				4.00	3.98	99.50
				6.00	6.12	102.00
				Mean ± SD		100.17±1.607
	THP	0.60	105.00 ± 1.169	0.40	0.397	99.25
				0.60	0.601	100.17
				0.80	0.816	102.00
				Mean ± SD		100.47±1.400

*Average of 6 determinations. ** Average of 3 determinations.
